# Mid-term results of total hip arthroplasty with anatomical ultra-short cementless stem in patients with developmental dysplasia of the hip Crowe type II

**DOI:** 10.1007/s00590-024-03844-7

**Published:** 2024-02-17

**Authors:** Maros Hrubina, Libor Necas, Marian Melisik, Zoltan Cibula, Peter Lisy, Juraj Cabala, Jozef Holjencik, Jozef Cabala

**Affiliations:** 1 Jessenius Faculty of Medicine in Martin, Comenius University in Bratislava, Martin, Slovak Republic; 2 University Department of Orthopaedic Surgery, University Hospital Martin, Kollarova 2, Martin, 036 59 Slovak Republic

**Keywords:** Total hip arthroplasty, Ultra-short anatomical cementless stem, Bony trabecular development, Developmental dysplasia of the hip

## Abstract

The aim of this study was to present the mid-term results of ultra-short cementless stem total hip arthroplasty (THA) in patients with Crowe type II developmental dysplasia of the hip. The study consists of 68 patients (75 THAs) with a Proxima stem implanted between 2006 and 2015. The clinical results include Harris Hip Scores. Radiological follow-up reports on stem migration, bony trabecular development and radiolucent lines. Kaplan–Meier survival analysis was performed. The mean age of patients was 48.4 years, with a mean follow-up 114 months. The average Harris Hip Score improved significantly from 45.1 preoperatively to 97.6 at the final evaluation (*p < *0.001). Stem migration was observed in five hips (in all of them up to the 6th postoperative month, without any further progression of migration or radiological loosening). Bony trabecular development was detected in modified Gruen zones (1, 2, 4, 6, 7 for Proxima stem): in zone 1 (0%), 2 (49.3.0%), 4 (38.7%), 6 (82.7%), 7 (0%). Radiolucent lines were observed around one cup (DeLee and Charnley zone I) and three stems (none was loose, all three with fibrous stable fixation). Complications were found in three hips (4.0%): intraoperative periprosthetic femoral fracture (threated with cerclage wire) in two hips and squeezing hip in one patient (with perioperative ceramic inlay breakage and exchange). No hip was revised. The implant survival was 100.0% both clinically and radiologically. Observations in the mean follow-up of 114 months show that the results (clinical and radiological) of the Proxima stem in patients with Crowe type II DDH are promising.

## Introduction

Total hip arthroplasty (THA) in patients with developmental dysplasia of the hip (DDH) is technically more challenging procedure due to distorted anatomy and altered biomechanics of the hip joint [1]. The dysplastic acetabulum is characterised by a small diameter, with deficient bony architecture, and proximalisation of the femoral head. The femoral challenges include a narrow medullary canal, excessive anteversion and high caput–collum–diaphyseal angle (CCD) with metadiaphyseal mismatch [2]. There are many studies reporting the results of various stems used in the treatment of secondary osteoarthritis due to DDH [3, 4]. Due to the fact that majority of patients with DDH are relatively young, the cementless stems should be considered. The complications of the conventional cementless stems are known: stress-shielding phenomenon, difficult revision, thigh pain, proximal femoral bone loss. To reduce these problems (by preservation of the femoral intramedullary canal and maintenance of femoral elasticity), ultra-short cementless anatomical stems were introduced [5]. The success of conservative femoral stems in selected patients has been published in the past [5–8]. There is a lack of reports of clinical studies of short stems in patients with DDH. One of the possible reasons is a very little freedom in intraoperative adjustment of the femoral anteversion depending on which modular stems are recommended, especially in Crowe types III and IV [9]. In patients with Crowe types I and II DDH, a short stem could provide good outcome, if performed meticulously. The aim of this study is to assess, both clinically and radiographically, a series of our patients who had undergone primary THA with the Proxima stem for the treatment of Crowe type II DDH, and who were followed for a minimum of eight years. We hypothesised that ultra-short anatomical stem THA would be a promising treatment option for patients with this type of secondary osteoarthritis in the mid-term.

## Materials and methods

Patients' records were reviewed retrospectively for demographic data, details of the surgery (implants used), X-rays and follow-up results.

The study group consisted of 68 patients (75 hips) with Crowe type II DDH [10]. All the patients were treated with primary THA (using the Proxima stem) between 2006 and 2015 at our University Department of Orthopaedic Surgery.

This study was approved by our institutional review board.

### Inclusion criteria

Included in this study were patients aged > 18 and < 65 years with good bone quality (Dorr type A or B) [11], with secondary arthritis due to Crowe type II DDH classification and with a minimum follow-up of 8 years.

### Exclusion criteria

Excluded were previously hip surgery (prior femoral osteotomy), trauma, oncologic or inflammatory diseases in the area of the affected hip, severe osteoporosis, femoral type Dorr C or incomplete follow-up.

Two hips with Proxima stem for DDH were excluded from the study because of incomplete follow-up.

### Surgical procedures

**Fig. 1:**
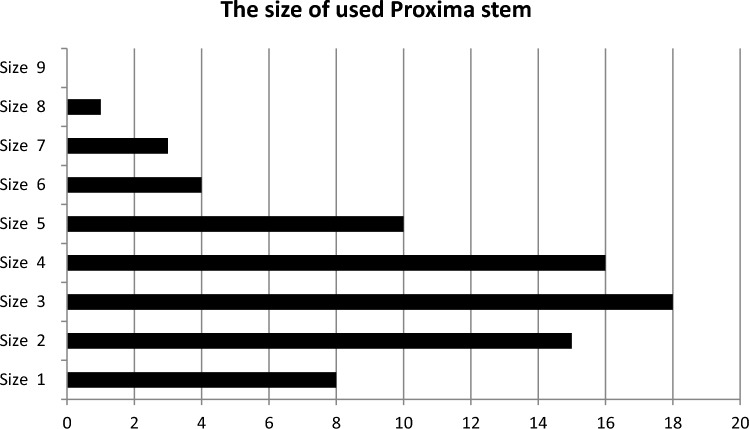
The sizes and numbers of used Proxima stems

Preoperative planning and templating on radiographs were used to determine the implant sizes [12]. All procedures were performed in the supine position, via an antero-lateral approach. The uncemented Pinnacle cup (DePuy, Warsaw, IN, USA), (range: 46–58 mm) was implanted with an lateral inclination of 40º–45˚ and 15˚ of anteversion, using a „press-fit“ technique following which ceramic inlay was inserted. Then, the ultra-short anatomical stem Proxima (DePuy, Leeds, UK) was inserted by the “round the corner” technique, followed the natural femoral neck anteversion [2]. After a trial reduction to assess hip joint stability and leg length discrepancy (LLD), the final stem was inserted. We used a standard stem in 70 cases and a high-offset stem in five cases; the most frequently used stem sizes were 3 (in 18 hips) and 4 (in 16 cases) (Fig. 1). The 28 and 36 mm ceramic heads were used according to the inlay diameter.

### Postoperative management

Active and passive movements were started on the first postoperative day. On the second postoperative day, patients were allowed to stand and were discharged 5 days postoperatively on average (range 4–8 days). Walking, touch-weight-bearing with crutches, was recommended until 6 weeks postoperatively (to prevent, early stem migration, rotational stress and possible loosening); at that stage patients were examined clinically and radiologically. Thereafter, partial weight-bearing was permitted. Full weight-bearing was allowed after the next examination, at the third postoperative month.

All patients were then followed clinically and radiologically at 6 months, 1 year and annually thereafter, until the end of follow-up period (during the year 2023).

### Clinical outcome measures

Basic demographic data were collected, including age, body mass index (BMI), Dorr type of femur. The Harris Hip Score (HHS) was assessed preoperatively and at final follow-up [13]. Thigh pain was assessed during physical examination. LLD was measured clinically and radiologically. Any early and late complications causing any potential need for the revision were recorded.

### Radiographic assessment

**Fig. 2:**
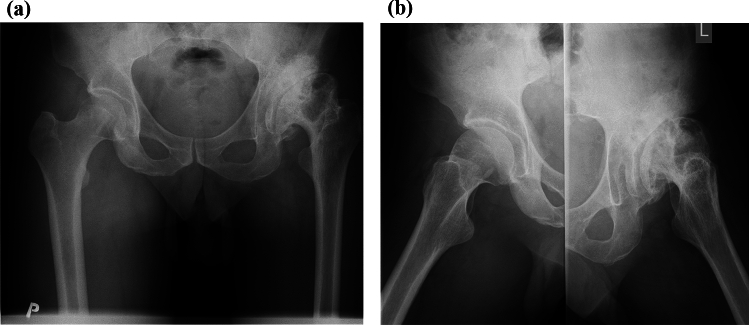
**a** Preoperative anteroposterior radiograph of 46-year-old patient with secondary postdysplastic coxarthrosis of the left hip joint (Crowe type II). The shortening of the affected extremity was of 2 cm. The deformity of the femoral head with the cysts is visible. **b** Preoperative axial radiograph of the same patient with postdysplastic coxarthrosis. The cyst in proximal part of the femoral head is visible

**Fig. 3:**
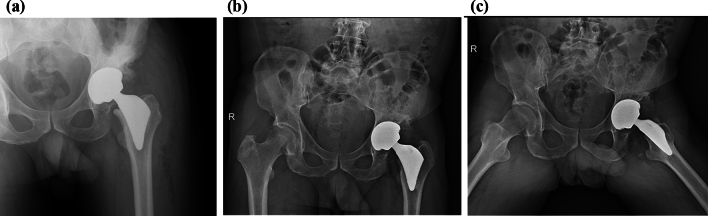
**a** Anteroposterior radiograph of the same patient on the first day after cementless total hip arthroplasty with the Proxima stem, placed in a neutral position (alignment). **b** Anteroposterior radiograph of the same patient, 12 years postoperatively, osseously stable stem fixation with bony trabecular development in modified Gruen zones 2 and 6 (around the distal part of the stem) and stress-shielding phenomenon in modified Gruen zone 7. **c**Axial radiograph of the same patient 12 years after surgery with visible bony trabecular development around the distal part of the stem

Preoperative, postoperative and final follow-up radiographs, in both orthogonal projections (anteroposterior and lateral), were evaluated blindly by an experienced orthopaedic radiologist. The Dorr type of the femur was assessed preoperatively, CCD was evaluated pre- and preoperatively. On the postoperative anteroposterior X-rays, the cup position (lateral inclination) and stem alignment (position) were evaluated. The stem position was assessed, according to Gombar et al. [6]: as “neutral”, if its deviation from the axis of the femoral shaft was 5˚ or less; “varus” or “valgus” with a deviation of 6°–10˚ and “severe varus” or “severe valgus” with greater deviation. Radiological evaluation during the follow-up period was focused on: stem migration, the presence of radiolucent lines around cup and stem, stress-shielding phenomenon and bony trabecular development in modified Gruen zones for the Proxima stem (zones 1, 2, 4, 6, 7), the “classic” Gruen zones 3 and 5 were not considered (Figs. 2a–3c) [14]. Stem migration was assessed according to Martel et al. [15]. Implant stability was evaluated based on the radiological features of the bone–implant interface, according to Engh et al. (osseously stable, fibrously stable or loose stems) [16]. Criteria for radiological loosening of the stem were defined as a radiolucent zone greater than 2 mm, or a migration greater than 3 mm with an adjacent radiolucent line surrounding the entire stem on the anteroposterior and lateral radiographs [5, 6].

Proximal femoral stress-shielding was evaluated according to Zicat et al. in modified Gruen zones for the Proxima stem [17]. Heterotopic ossification was assessed according to Brooker’s classification [18].

### Statistical analysis

All statistical analyses were performed using R software version 4.0.2 (R Core Team 2020). Continuous variables were expressed as mean and standard deviation (SD), as well as range. Dichotomous outcomes were expressed as the number of events and percentage. The normality of the HHS data was confirmed using the Shapiro–Wilk test (*p < *0.05).

Two-sided, paired Student’s *t* test was used for statistical analysis of the pre- and postoperative HHSs. Statistical differences were considered to be significant when the *p* value was < 0.05. Relatively large effects (following the guidelines from Cohen, 1988) were reported in the previous studies for HHSs. A power analysis, using the R 3.5.0 (power package), indicated that a total sample of 15 patients-hips would be needed to detect large effects (*d* = 0.8) with 80% power, using a two-sided paired *t* test between means with alpha at 0.05 [19].

Survivorship analysis was performed with the Kaplan–Meier estimator with the end point at 114 months.

## Results

### Clinical analysis

All 68 patients (75 hips) were followed-up and the final evaluation was performed at a mean of 114.0 months (range 98–188 months, standard deviation [SD] 33.7 months).

There were seven cases with bilateral (second implantation was performed 4–13 months after the first THA) and 61 hips with unilateral procedures. Fifty-nine patients were women and nine patients were men. The mean age of the patients was 48.4 years (18–66 years, SD 9.5 years). The mean BMI was 27.7 kg/m^2^ (17.20–42.96, SD 4.4 kg/m^2^) (Table 1). The mean preoperative HHS improved significantly from 45.2 (19–65, SD 10.8) to 97.7 (78–100, SD 4.3) at final follow-up (*p* = 0.001). Finally, 40 patients (58.8%) had excellent hip scores (range 90–100 points), 27 patients (39.7%) had good scores (range 80–89 points), one patient (1.5%) had fair score (range 70–79 points), and no patient had a poor score (less than 70 points).

**Table 1:** Patients' demographic and details of the group with the Proxima stem for dysplastic hips

Parameters	Values
No. of hip arthroplasties (unilateral–bilateral)	61/7
Gender (male/female)	9.51
Mean age (years, range, SD)	48.4 (18–66, SD 9.5)
Mean follow-up (months, range, SD)	114 (98–188, SD 33.7)
Mean BMI kg/m^2^ (range, SD)	27.7 (17.20–42.96, SD 4.4)
*Dorr classification (cases)*	
A	43
B	32
*CCD angle (degrees, range, SD)*	
Preoperative	146.6 (128–162, SD 5.2)
Postoperative	133.4 (122–142, SD 3.4)
*LLD (mm, range, SD)*	
Preoperative	26 (5–54, SD 26)
Postoperative	4 (0–14, SD 8)
*Harris Hip Score*	
Mean preoperative (points, range, SD)	45.2 (19–65, SD 10.8)
Mean at final evaluation (points, range, SD)	97.7 (78–100, SD 4.3)

LLD was 2.6 cm (average shortening) before the operation (range 0.5–5.4 cm, SD 2.6 cm) and 0.4 cm (average elongation of the operated extremity) at final follow-up (range 0.0–1.4 cm, SD 0.8 cm).

Thigh pain was observed in one patient with a fair score and a fibrous stable stem (1.5%).

### Radiographic analysis

All evaluated hips were Crowe type II dysplastic hips. Dorr type A femoral form was found in 43 hips (57.3%); Dorr type B was found at 32 hips (42.7%). No femur of Dorr type C was found. The initial cup position (lateral inclination) was on average 46° (range 40°–58°, SD 5.2°) without cup migration during the whole follow-up. We found a radiolucent line of 1 mm width in one cup (clinically asymptomatic) in DeLee–Charnley zones I–III [20].

This radiolucent line appeared on X-ray at 3rd postoperative month and was without progression at the last visit (14th postoperative year). The postoperative stem alignment was evaluated as normal in 64 hips (85.3%), and varus (to 10°) in 11 hips (14.7%). Migration (subsidence and varisation) was observed in five stems (6.7%).

Stem fixation, using the criteria of Engh et al., was osseously stable in 72 hips (96.0%), and fibrously stable in three stems (4.0%). No stem was loose.

Radiolucent lines (in modified Gruen zones 4, 6 and 7) were observed only in three fibrously stable stems: two of these patients remained pain free, one had thigh pain. These stems exhibited fibrous stability, with the subsidence up to 2 mm and varisation up to 10° until the 6th postoperative month, without any further progression during the whole follow-up period (9th, 12th and 15th year).

Another two stems (in unilateral hips) showed migration up to 2 mm with varisation to 10° during the first six postoperative months without any other changes—these stems were considered as osseously stable.

Stress-shielding grade I, according to Zicat et al. in modified Gruen zone 7, was found in 72 stems (96.0%); this phenomenon was not found in fibrously stable stems at the final follow-up.

Heterotopic ossification was diagnosed in six hips (8.0%)—Brooker type I was found in five patients, and type II in one.

Bony trabecular development was found in all 72 osseously stable stems (Table 2) and their location evaluated in modified Gruen zones: in zone 2 in 51 hips (68.0%), in zone 4 in 47 hips (62.7%), in zone 6 in 48 hips (64.0%).

**Table 2:** Bony trabecular development and radiolucent lines around the Proxima stem in modified Gruen zones 1, 2, 4, 6, 7

Zone (modif. Gruen)	Bony trabecular development *n*(%)	Radiolucent lines *n*(%)
1	0	0
2	51 (68.0%)	0
4	47 (62.7%)	3 (4.0%)
6	48 (64.0%)	3 (4.0%)
7	0	3 (4.0%)

In patients with fibrously stable stems, we found no development of trabecular bone.

The mean CCD angle was 146.6° (range 128°–162°, SD 5.2°) preoperatively, the mean CCD angle postoperatively was 133.4° (range 122°–142°, SD 3.4°).

There were not any different findings in results in cases with bilateral hips compared to cases with unilateral procedures.

### Complications and revisions

Complications were observed in three hips out of 75, with a unilateral Proxima stem (4.0%), without the need for revision surgery.

Intraoperative fracture of the ceramic inlay was observed in one hip. The broken inlay was removed and a new one was inserted. Ten years after the surgery, the patient is pain free and clinically satisfied, but with the squeezing phenomenon occurring four years after the surgery. The implant showed no signs of loosening, or insert breakage, and the stem was osseously stable.

In two hips, an intraoperative femoral neck crack occurred. Both were treated with cerclage wiring, without any further complications during follow-up. The patients were pain free and clinically satisfied during their last visits (124- and 162-month follow-up), the implants showed no signs of loosening and the stems were osseously stable.

Kaplan–Meier survival analysis indicated a clinical survival rate of 100.0%, with a radiological survival rate of 100.0%.

Survival for revision for aseptic loosening was 100% in our group of patients.

## Discussion

The excellent results of the short stem cementless THA in patients with primary osteoarthritis, or osteonecrosis of the femoral head (ONFH), have been published [5–8, 21].

The success of non-cemented THA relies on osteointegration. The short stems have been introduced for the surgery in young patients with the intention of reducing the risk of periprosthetic bone resorption, thigh pain and to preserve as much bone as possible for any further revision [5]. The disadvantages of these stems are: possible stem malposition in a narrow femoral canal; under-sizig, which can lead to inadequate early stability, with possible loosening of a fibrous stable fixation [22]. Due to extensive experience and good clinical results in patients treated with the use of Proxima stem in primary osteoarthritis and ONFH, we started to use this stem in dysplastic hips. Crowe type II DDH is the upper limit, above which the short stems should not be used [2]. Severely dysplastic hips (Crowe type III and IV) should be treated with modular stems that allow the possibility of anteversion correction perioperatively [23].

There are not many studies about the use of short stems in secondary osteoarthritis due to DDH.

Budde et al. evaluated results in 58 Metha stems in DDH. The HHS significantly increased and CCD decreased. The revison’s rate was 1.7%, but only two patients had Crowe type II deformity [23].

Suksatien et al. analysed results of the same stem in 32 dysplastic hips. Nineteen cases were Crowe type II DDH. The mean age of these patients was 50.3 years, the mean follow-up period was 77 months. The average HHS increased from 30.3 preoperatively to 93.6 at the last follow-up. The Kaplan–Meier stem survivorship was 100%. Bony trabecular development was observed in Gruen zone 1 in 93.9%, in Gruen zone 2 in 93.9%, in Gruen zone 3 in 25%, in zone 5 in 6.3%, in zone 6 in 96.9% and in zone 7 in 90.6%, but the Metha implant has a different design from the Proxima. They did not observe periprosthetic fracture, or stem loosening, leading to the need for revision [2].

Drosos et al. reported the results of the Minima short cementless stem. From the 61 patients, only 13 (21.6%) THAs were performed due to DDH, without the specification of the Crowe type. The average HHS increased from 58.7 preoperatively to 95.1 at the last follow-up (mainly 3 years). The Kaplan–Meier stem survivorship was 100%. All stems were osseously stable at the final examination. Reactive cortical hypertrophy was observed in four of the Minima stems [22].

Melisik et al. presented a study dealing with the Proxima stem, in which the incidence of secondary postdysplastic osteoarthritis was 30% (39 cases of 130). They reported radiological survival of 98.5% and clinical survival of 100% after an average 10 years in whole group of patients, with a mean age of 45.5 years. The average HHS improved from 42.5 preoperatively to 98.8 at the final evaluation. Similarly to our study, the authors reported mild migration and varisation in 23 stems (17.7%), which settled without any further progression by the 6th postoperative month. Radiolucent lines were observed around five stems (3.8%) [14].

Gombar et al. presented results of 86 hips with the Proxima stem in patients with an average age of 50 years. The incidence of postdysplastic (mild to moderate DDH) osteoarthritis was 9%. The average HHS improved from 40 to 91 by the final evaluation. They reported intraoperative periprosthetic fractures in three hips (3.5%), with the need for revision in one. Stem malalignment was observed in 10 hips (12%). Stem subsidence with osteolysis was observed in one case with undersizing. The overall stem survival was of 97% after 7 years. In our study, we have reported two cases of intraoperative periprosthetic fractures treated with cerclage wires, without any other problems [6].

Kim et al. published a study of the 324 hips in patients younger than 50 years, with a Proxima stem. Thirty-four per cent (81 patients) of cases were primary dysplastic hips. The femoral stem survival was 99.1% after an average 15.6 years. They reported primary varus position (more than 5°) of the stem in 6% of stems. 0.9% of stems (*n* = 3) were revised for aseptic loosening, in two hips they recorded early periprosthetic fracture. The average HHS improved from 37 to 93 points at the final follow-up [5].

Mahmoud et al. assessed a migration pattern (subsidence and malalignment—varisation of the Proxima stem). We have found this phenomenon in two stems, the migration stabilised after the sixth postoperative month [24].

Uemura et al. presented results of the anatomical short stem CentPillar. From the 222 hips, the DDH Crowe type II was presented in 32 cases. The overall stem survival was of 99% after median follow-up 13.1 years. They performed two stem revisions (one for aseptic loosening and one for late infection) [25].

Bony trabecular development around the Proxima stem has rarely been described. It occurred around the distal half of the stem (modified Gruen zones 2–6) and when this phenomenon occurred, the stem was osseously stable [14]. Our study revealed similar results.

From the above mentioned studies, it follows that the short femoral stem survivorship in patients with DDH is in the range from 97 to 100% at 10-year follow-up, with the use of different short stems [5, 6]. Our results are within this range.

The patients with bilateral and unilateral hip dysplasia were included both, due to the fact that no patient had bilateral simultaneous THA. The difference between these patients did not affects the results. The complications occurred only in unilateral hips.

Similar to the published studies, we have observed significantly improved function of the hip, as evaluated using HHS. Bony trabecular development was observed mainly around distal part of the stem in our study—in modified Gruen zones 2, 4 and 6. This indicates the physiological load transfer through the proximal femur, as also shown by bone mineral density analysis in the study of Kim et al. [5]. We have observed stress-shielding phenomenon around osseously stable Proxima stems in zone 7, as was also reported by other authors [4–6, 8, 14]. Initial varisation and subsidence, without progression after the 6th postoperative month, had not led to implant loosening at the last visit.

Our study has some limitations. Firstly, this study was retrospective in design, not randomised and used no control group, without which it is not possible to draw any direct conclusion on the performance of the implant in question relative to any other. Secondly, this study was not based on dual-energy radiograph absorptiometry, and so we were not able to assess exactly changes in bone density.

However, the study provides relative long follow-up, and no patient was lost.

In conclusion, we report that primary THA in patients with DDH Crowe type II with the use of anatomical cementless Proxima stem has promising outcomes in the mid-term. This stem design enables the preservation of the proximal femoral bone.

## Data Availability

Data and material available on request.
